# Control over Electrochemical CO_2_ Reduction Selectivity by Coordination Engineering of Tin Single‐Atom Catalysts

**DOI:** 10.1002/advs.202102884

**Published:** 2021-10-24

**Authors:** Jiangyi Guo, Wenlin Zhang, Lu‐Hua Zhang, Datong Chen, Jiayu Zhan, Xueli Wang, N. Raveendran Shiju, Fengshou Yu

**Affiliations:** ^1^ Tianjin Key Laboratory of Chemical Process Safety National‐Local Joint Engineering Laboratory for Energy Conservation in Chemical Process Integration and Resources Utilization School of Chemical Engineering and Technology Hebei University of Technology Tianjin 300130 P. R. China; ^2^ Van’t Hoff Institute for Molecular Sciences University of Amsterdam P.O. Box 94157 Amsterdam 1090GD The Netherlands

**Keywords:** asymmetric SnN_3_O_1_ configuration, CO selectivity, CO_2_ reduction reaction, electrochemistry, single‐atom catalysts

## Abstract

Carbon‐based single‐atom catalysts (SACs) with well‐defined and homogeneously dispersed metal−N_4_ moieties provide a great opportunity for CO_2_ reduction. However, controlling the binding strength of various reactive intermediates on catalyst surface is necessary to enhance the selectivity to a desired product, and it is still a challenge. In this work, the authors prepared Sn SACs consisting of atomically dispersed SnN_3_O_1_ active sites supported on N‐rich carbon matrix (Sn‐NOC) for efficient electrochemical CO_2_ reduction. Contrary to the classic Sn‐N_4_ configuration which gives HCOOH and H_2_ as the predominant products, Sn‐NOC with asymmetric atomic interface of SnN_3_O_1_ gives CO as the exclusive product. Experimental results and density functional theory calculations show that the atomic arrangement of SnN_3_O_1_ reduces the activation energy for *COO and *COOH formation, while increasing energy barrier for HCOO* formation significantly, thereby facilitating CO_2_‐to‐CO conversion and suppressing HCOOH production. This work provides a new way for enhancing the selectivity to a specific product by controlling individually the binding strength of each reactive intermediate on catalyst surface.

## Graphical abstract

A Sn‐based single‐atom catalyst with asymmetric atomic interface of SnN_3_O_1_ is designed and synthesized to switch the product of CO_2_ reduction from HCOOH to CO with an impressive FE_CO_ of 94%.

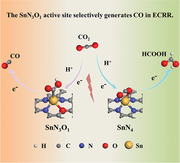

## 1 Introduction

Electrochemical CO_2_ reduction reaction (ECRR) has been recognized as one of the promising strategies to meet the CO_2_ abatement goal of the world by converting it to value‐added products.^[^ ^1^ ^]^ However, ECRR usually involves the multiple proton‐coupled electron transfer (PCET) steps and competes with the hydrogen evolution reaction (HER) causing poor selectivity and lower conversion efficiency. Designing catalysts with well‐defined and homogeneously dispersed active centers can reduce the diversity of CO_2_ reaction routes and weaken HER, thus improving selectivity to a specific product at low potentials.

Recently, carbon‐based single‐atom catalysts (SACs) with isolated single metal–N*_x_* (M–N*_x_*) centers have emerged as new candidates for ECRR electrocatalysts.^[^ ^2^ ^]^ The defined structure and coordination environment provide a great opportunity to tune adsorption behavior of substrate and intermediates, thus controlling the reaction pathways for efficiently and selectively producing a desired product.^[^ ^3^ ^]^ Tin (Sn) SACs are generally regarded as catalysts for converting CO_2_ to formic acid owing to their moderate adsorption energy for HCOO* intermediate.^[^ ^4^ ^]^ However, the currently reported SnN_4_ structure exhibits poor selectivity for ECRR. For instance, Wei et al. reported a SnN_4_ configuration embedded on a hierarchical integrated carbon nanosheet array showing 70% Faraday efficiency (FE) for HCOOH production with around 15% CO and 15% H_2_ formation.^[^ ^5^ ^]^ The low selectivity was induced by the tiny thermodynamical difference in energy barriers for HCOOH, CO, and H_2_ formation paths. Therefore, controlling the binding strength of various reactive intermediates on catalyst surface is necessary to enhance the selectivity to a desired product, and it is still a challenge.

Recent research has revealed that introducing the second coordination heteroatom can cause significant differences in the binding energy of active centers to intermediates, subsequently leading to a change in the reaction routes. For instance, a series of Fe_1_N_4_–O_1_ (Fe_1_N_4_–O_1_ site with axial Fe–O coordination),^[^ ^6^ ^]^ Cu‐SN_3,_^[^ ^7^ ^]^ Mn‐N_4_Cl,^[^ ^8^ ^]^ Ni‐N_3_S,^[^ ^9^ ^]^ and Fe‐N_3_S^[^ ^10^ ^]^ configurations have been reported and shown higher catalytic performance than symmetrical M–N_4_ structure under the same conditions. In spite of the progress, the research about the effect of asymmetrically coordinated Sn single‐atom sites on reaction pathways of ECRR is limited. Moreover, the determination of real active sites and how these single‐atom sites boost ECRR are still not well understood.

In this work, we designed and synthesized Sn SACs consisting of atomically dispersed SnN_3_O_1_ active sites supported on N‐rich carbon matrix (Sn‐NOC), which displayed exceptional activity for the ECRR to CO with a maximum FE of 94% and a CO partial current density of 13.9 mA cm^−2^ at −0.7 V (versus RHE). In contrast, for the reference tin phthalocyanine (Sn‐Pc) with classic Sn‐N_4_ configuration, HCOOH and H_2_ products dominated over the measured potential range. According to the number of active Sn atoms in Sn‐NOC, the calculated TOF of ECRR to CO is 23 340.5 h^−1^, which is more than 400 times over that of Sn‐N_4_ (57.5 h^−1^) and superior over the advanced SACs in literature. Moreover, a linear relationship is established by plotting the FE_CO_ versus the Sn‐N content, proving that SnN_3_O_1_ coordination is the real active site for ECRR in the Sn‐NOC catalyst. Experimental results and density functional theory (DFT) calculations show that the SnN_3_O_1_ configuration has an optimal adsorption capacity for *CO_2_ and *COOH intermediates, which is beneficial to the generation of CO, shifting the production from HCOOH to CO. Our finding offers a universal strategy for enhancing the catalytic performance of SAC and controlling its selectivity.

## 2 Results and Discussion

**Figure 1:**
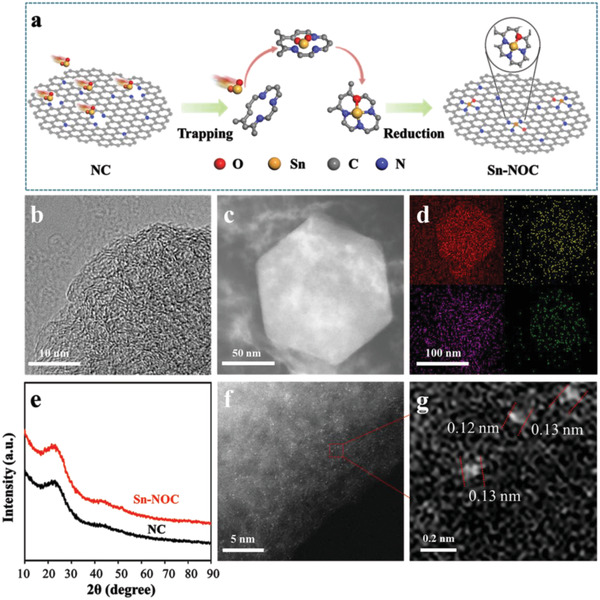
a) The formation process of atomically dispersed SnN_3_O_1_ active sites; b) HRTEM image, c,d) STEM images, and corresponding EDS mapping of Sn‐NOC; e) XRD patterns of NC and Sn‐NOC; f,g) AC HAADF‐STEM images of Sn‐NOC.

The atomically dispersed SnN_3_O_1_ active sites supported on N‐rich carbon matrix (NC) were synthesized by a gas transport strategy (**Figure** **1**a).^[^ ^11^ ^]^ NC was obtained by a simple pyrolysis of ZIF‐8 precursor. SnO_2_ powder, serving as the Sn and O sources, and NC were subsequently placed in two separate porcelain boats and were heated at high temperatures. In high temperature Ar flow, volatile Sn species, formed by the evaporation of SnO_2_, migrated to the surface of NC and were trapped by heteroatoms in carbon basal plane, forming the isolated Sn‐NOC electrocatalyst. Notably, the relative contents of Sn atoms in Sn‐NOC samples can be controlled by varying the temperature from 950, 1000 to 1050 °C. The samples are denoted as Sn‐NOC‐950, Sn‐NOC‐1000 (i.e., Sn‐NOC), and Sn‐NOC‐1050, respectively. This provides a possible way to correlate catalytic activity with the amounts of active sites, thereby identifying the real active sites for ECRR. This will be discussed in detail below.

Transmission electron microscopy (TEM) images show that both NC and Sn‐NOC (Figure S1, Supporting Information) retained the dodecahedral morphology of ZIF‐8 without obvious surface distortion and structural collapse, and no obvious metal nanoparticles were observed. High‐resolution TEM (HRTEM) image reveals that the microstructures of Sn‐NOC sample have massive crystallites stacked by graphitic layers (Figure 1b). Raman spectra of the two samples show two obvious peaks near 1580 and 1348 cm^−1^ corresponding to the stretching vibrations of sp^2^‐bonded (G‐bond) carbon and defect‐induced breathing mode of aromatic ring (D bond), further aligned with graphitic carbon structure (Figure S2, Supporting Information). The energy‐dispersive X‐ray spectroscopy (EDS) mapping research shows that the C, O, N, and Sn were dispersed evenly throughout the Sn‐NOC (Figure 1c,d). X‐ray diffraction (XRD) of the two samples show similar diffraction patterns with broad peaks located at 23° and 44° assigned to the (002) and (101) planes of graphitized carbon (Figure 1e).^[^ ^12^ ^]^ No obvious metallic Sn crystallites were detected, consistent with the results of HRTEM. For revealing the dispersion of Sn species more clearly, we also did the aberration‐corrected high‐angle annular dark‐field scanning TEM (AC HAADF‐STEM) and the images are shown in Figure 1f,g. The individual bright dots with a size of ≈0.13 nm are consistent with the size of Sn atom, validating that the Sn species are atomically isolated on the carbon matrix.^[^ ^13^ ^]^ The Sn contents in Sn‐NOC‐950, Sn‐NOC‐1000, and Sn‐NOC‐1050, analyzed by inductively coupled plasma atomic emission spectrometry (ICP‐MS), are 0.27, 0.48, and 0.43 wt.%, respectively.

**Figure 2:**
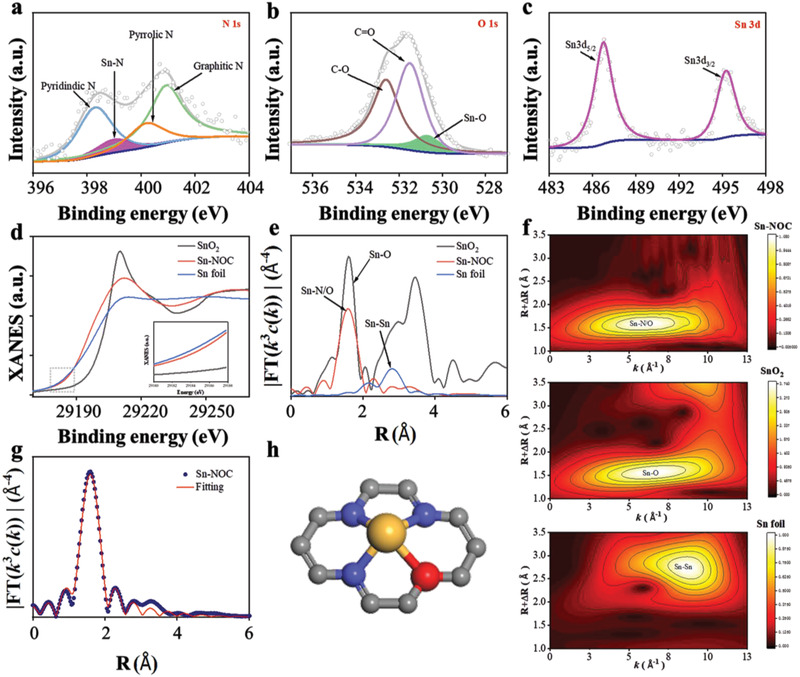
a–c) XPS spectra of N1s, O1s, and Sn3d of Sn‐NOC; d) Sn K‐edge XANES spectra; e) Fourier transform (FT) of the Sn K‐edge EXAFS spectra; f) WT analysis of Sn‐NOC, SnO_2_, and Sn foil; g) The fitting curve of k^3^‐weighted EXAFS spectra; h) Schematic atomic interface model of Sn‐NOC.

The chemical composition and elemental states of NC and Sn‐NOC were analyzed by X‐ray photoelectron spectroscopy (XPS, Figures S3 and S4 and Table S1, Supporting Information). High‐resolution N1s spectrum shows distinct Sn‐N bond at ∼399.0 eV, suggesting the direct coordination of Sn and N atoms (**Figure** **2**a).^[^ ^14^ ^]^ Additionally, pyridine N (398.4 eV), pyrrole N (400.1 eV), and graphite N (401.4 eV) were also detected.^[^ ^15^ ^]^ The high‐resolution O1s spectrum consists of three sub‐peaks, C═O (531.1 eV),^[^ ^16^ ^]^ C–O (532.1 eV),^[^ ^17^ ^]^ and Sn–O (530.7 eV) (Figure 2b), implying Sn is also trapped by O atom. For Sn 3d spectrum of Sn‐NOC, the Sn 3d_5/2_ peak centers at 486.5 eV, higher than Sn^0^ (484.5–485.5 eV) and lower than Sn^4+^ (486.3–487.3 eV) indicating the valence of Sn is 0 < *δ* <4 (Figure 2c).^[^ ^18^ ^]^

To further explore the chemical states and coordination environment of the atomically dispersed Sn atoms, Sn‐NOC was further characterized by synchrotron‐radiation‐based X‐ray absorption spectroscopy (XAS, consisted of X‐ray absorption near‐edge structure (XANES) and extended X‐ray absorption fine structure (EXAFS)). The Sn K‐edge XANES shows that the oxidation state of the isolated Sn atoms in Sn‐NOC is higher than Sn^0^ and lower than Sn^4+^ (Figure 2d), consistent with XPS analysis. The oxidation state of isolated Sn atom was further analyzed and found as +1 by the Linear Combination Fitting (LCF) of the XANES data (Figure S5 and Table S2, Supporting Information). The pre‐edge features of the XANES spectra of Sn‐NOC show a clear shift towards the left compared to that of Sn foil, indicating that Sn atoms in Sn‐NOC can easily transfer electrons and interact with substrate, for example, CO_2_. The FT‐EXAFS curve of Sn‐NOC shows a main peak at around 1.51 Å assigned to the Sn–N or Sn–O coordination (Figure 2e).^[^ ^5^ ^]^ Importantly, the characteristic peak of Sn–Sn bond at about 2.77 Å was not detected, confirming the isolated Sn atoms on the carbon skeleton. We also used wavelet transform (WT) to analyze the Sn K‐edge EXAFS oscillations. The WT maximum for the Sn‐NOC was found at 6.2 Å^−1^ which could be assigned to the Sn–N/O. No signals assigned to Sn–Sn were detected as compared with WT plots for Sn foil and SnO_2_ (Figure 2f). Based on the subsequent EXAFS fitting with the bond distances of Sn–N and Sn–O, the local atomic configuration of Sn atom in Sn‐NOC is consistent with the SnN_3_O_1_ model (Figure 2g,h and Table S3, Supporting Information).

N_2_ sorption isotherms for the two samples are essentially type I, indicating their microporous nature (Figure S6 and Table S4, Supporting Information). Remarkably, the pore volumes and surface areas of Sn‐NOC have significantly increased compared with those of NC, meaning more physical adsorption sites for CO_2_. In addition, a strong CO_2_ chemisorption capacity of Sn‐NOC was confirmed by CO_2_ temperature‐programmed desorption (CO_2_‐TPD) analysis (Figure S7, Supporting Information).^[^ ^19^ ^]^

**Figure 3:**
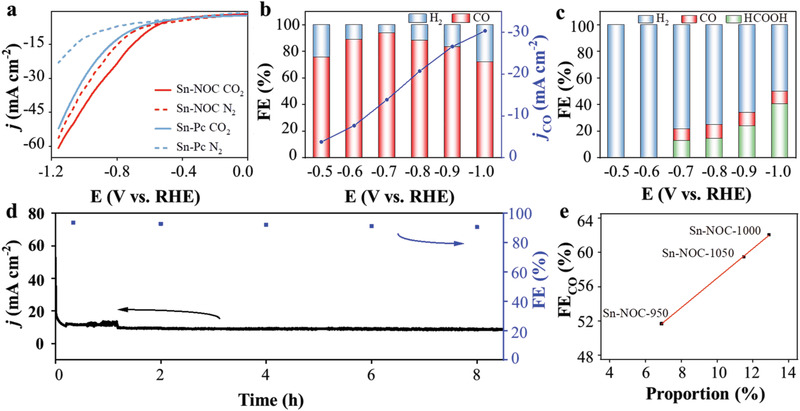
a) LSV curves of Sn‐NOC and Sn‐Pc in CO_2_‐saturated or N_2_‐saturated 0.1 M KHCO_3_ solution; b) FE and partial current densities of Sn‐NOC at various potentials in a typical H‐type cell; c) FE of Sn‐Pc at various potentials; d) Chronoamperometry curve and FE_CO_ by Sn‐NOC at −0.7 V in CO_2_‐saturated 0.1 M KHCO_3_ aqueous solution; e) The linear relationship between Sn‐N content and FE of Sn‐NOC catalysts prepared at different temperatures.

To test activity and selectivity of the samples for ECRR, linear sweep voltammetry (LSV) analysis was first carried out in a three‐electrode H‐cell containing CO_2_‐ or N_2_‐saturated 0.1 M KHCO_3_ solution. As shown in **Figure** **3**a and Figure S8, Supporting Information, Sn‐NOC shows higher reduction current density starting at −0.5 V in the CO_2_‐saturated electrolyte than in the N_2_‐saturated electrolyte, implying an active response to ECRR. Moreover, this current density is higher than that of tin phthalocyanine (Sn‐Pc) with a classic SnN_4_ configuration, and that of NC in CO_2_‐saturated electrolyte. Impressively, the current density of Sn‐NOC at −0.7 V could reach 14.81 mA cm^−2^, which is much higher than Sn‐Pc (8.42 mA cm^−2^) and NC (8.44 mA cm^−2^). The FE was evaluated through controlled potential electrolysis with the detection of the products by gas chromatography and ion chromatography. For Sn‐NOC, CO was detected as the main reduction product, and FE_CO_ reaches 94% at −0.7 V. A high selectivity of more than 80% can be retained at the potential range of −0.6∼−0.9 V (Figure 3b). In contrast, Sn‐Pc, a control sample, exhibits a very low selectivity for CO (FE_CO_ < 10%), while HER dominates with FE higher than 50.0% at the measured potential range (Figure 3c). Similarly, SnO_2_ nanoparticles loaded on NC (denoted as SnO_2_‐NC)^[^ ^20^ ^]^ also showed low CO selectivity and produced more HCOOH (Figures S9–S11, Supporting Information). The impressive selectivity for CO and excellent activity over a wide potential range enhances the CO partial current density (*j*_CO_) for Sn‐NOC, reaching 26.6 mA cm^−2^ at −0.9 V, which is 13.4 and 4.2 times more than that for Sn‐Pc and NC, respectively (Figure S12, Supporting Information). The Tafel slope derived from CO partial current density was 110 mV dec^−1^ for Sn‐NOC, lower than that of NC (129 mV dec^−1^) and Sn‐Pc (281 mV dec^−1^) (Figure S13, Supporting Information), further illustrating that the kinetics enhancement of Sn‐NOC. The Tafel slope of Sn‐NOC (110 mV dec^−1^) is close to 118 mV dec^−1^ indicating that the rate‐determining step (RDS) is the initial electron transfer to CO_2_ forming *COOH intermediate. Additionally, a stable current density with FE_CO_ more than 90% was achieved for 8 h by Sn‐NOC catalyst (Figure 3d). To explore the intrinsic activity of the as‐prepared catalysts, the catalytic current was normalized with electrochemically active surface area (ECSA) extracted from double‐layer capacitance (*C*_dl_). The Sn‐NOC catalyst showed a *C*_dl_ of 10.03 mF cm^−2^ which is higher than NC (5.18 mF cm^−2^, Figure S14, Supporting Information), indicating more ECRR active sites were exposed by the introduction of Sn atoms. The specific activity normalized to the measured ECSA of Sn‐NOC is also more than that of the control samples (Figure S15, Supporting Information), further suggesting a high intrinsic catalytic activity of Sn‐NOC for CO production. According to the number of active Sn atoms, the calculated TOF of ECRR to CO is 23 340.5 h^−1^, which is more than 400 times over that of Sn‐N_4_ (57.5 h^−1^) and superior over the advanced SACs reported in literature (Tables S5 and S6, Supporting Information), reflecting a highly enhanced intrinsic activity.

To further clarify the active sites of Sn‐NOC in ECRR, thiocyanate (SCN^−^) poisoning experiments were carried out.^[^ ^21^ ^]^ Generally, SCN^−^ can adsorb on the M‐N*_x_* moiety leading to the loss of catalytic activity, while SCN^−^ shows no obvious poisoning effect for heteroatom active sites, that is, N. Therefore, we prepared a series of Sn‐NOC samples with different contents of single atomic Sn active sites by controlling the temperature of Ar flow at 950, 1000, and 1050 °C, respectively. During ECRR test, 10 mM KSCN was added to 35 mL CO_2_‐saturated 0.1 M KHCO_3_ aqueous solution. As shown in Figures S16–S20, Supporting Information, Sn‐NOC‐1000 sample shows the highest conversion ability for CO_2_ to CO over the measured potential range. Notably, a linear relationship is established by plotting the FE_CO_ versus the Sn‐N content, proving that atomically dispersed SnN_3_O_1_ coordination is the real active site for ECRR in the Sn‐NOC catalyst (Figure 3e, Figure S21 and S22, and Table S7, Supporting Information).

**Figure 4:**
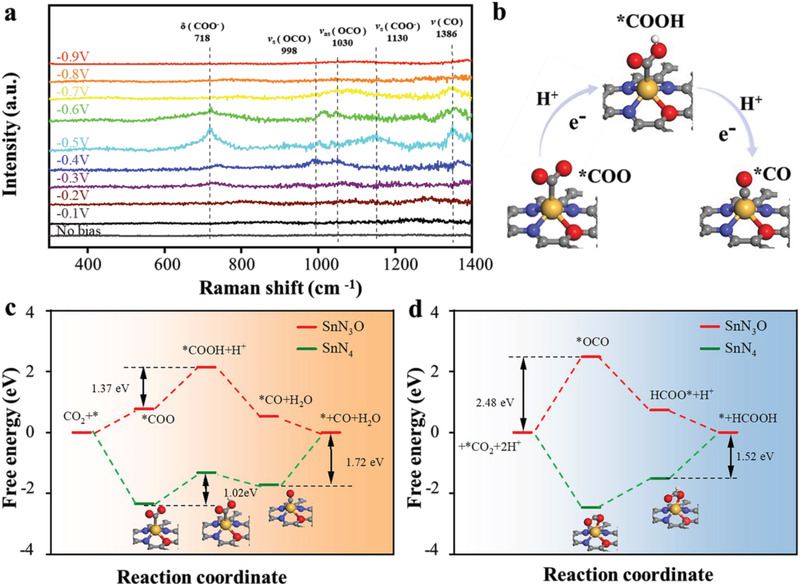
a) In situ SERS spectra of ECRR at the Sn‐NOC surface in 0.1 M CO_2_‐saturated KHCO_3_. b) The formation of *COO^−^ and *COOH pathway on Sn‐NOC. c) The calculated Gibbs free energy diagrams for CO_2_‐to‐CO conversion. d) The calculated Gibbs free energy diagrams for CO_2_‐to‐HCOOH conversion.

To our best knowledge, there are limited studies on the identification of the real active sites in N‐rich carbon catalyst for ECRR. To identify possible reaction intermediates leading to fundamental understanding of the ECRR mechanism, we conducted electrochemical in situ surface‐enhanced Raman spectroscopy (SERS) under different potentials (**Figure** **4**a). Four peaks (718, 998, 1030, and 1130 cm^−1^) were observed from −0.3 to −0.6 V which can be assigned to the *δ* (COO^−^) from *COO^−^, the symmetric stretch of *OCO, the asymmetric stretch of *OCO, and the stretch of *CO from *COOH, respectively.^[^ ^22^ ^]^ The signal changes from weak to strong and then weak with the increase of potential from −0.1 V to −0.9 V, and a plateau appears at −0.5 V consistent with potential trend in activity evaluation, indicating the formation of *COO^−^ and *COOH pathway on Sn‐NOC (Figure 4b). As the applied voltage increases, the driving force becomes larger and the reaction rate increases, resulting in rapid consumption of the intermediate and therefore weak signals in SERS at large FE.

Subsequently, DFT calculations were carried out to explore the catalytic mechanism of SnN_3_O_1_ for ECRR (Figures S23 and S24, Supporting Information). The free energy diagram (Figure 4c) shows that the ECRR on SnN_4_ site is controlled by the desorption of *CO with Δ*G* = 1.72 eV.^[^ ^23^ ^]^ The *COO adsorption is facile on SnN_4_. However, it is an endothermic process on SnN_3_O_1_, followed by an uphill step to form *COOH as the RDS consistent with Tafel plot analysis. SnN_3_O_1_ exhibits a relatively lower energy barrier at the RDS (Δ*G* = 1.37 eV), that is, *COOH formation than that of SnN_4_ (Δ*G* = 1.72 eV, the step of *CO desorption), resulting in an enhanced CO generation. In the case of CO_2_‐to‐HCOOH (Figure 4d), *OCO is spontaneously adsorbed on the SnN_4_ position, and the processes of HCOO* formation and HCOOH desorption are endothermic.^[^ ^24^ ^]^ HCOOH desorption is the RDS with Δ*G* = 1.52 eV for SnN_4_. In contrast, *OCO adsorption is energy‐intensive and the required energy barrier is as high as 2.48 eV on the SnN_3_O_1_, therefore inhibiting the HCOOH formation significantly. Therefore, the asymmetric atomic interface of SnN_3_O_1_ facilitates *COO and *COOH coordination and boosts CO formation with the suppression of HCOOH formation. Additionally, from the kinetic point of view, the asymmetric atomic interface facilitates the electron transfer and therefore promotes the protonation process of CO_2_, ultimately accelerating the formation of CO.^[^ ^7^ ^]^ Additionally, SnN_3_O_1_ increases the *H endothermic energy of HER, which is the main competitive reaction of ECRR (Figure S25, Supporting Information).

## 3 Conclusion

We demonstrate that a Sn single‐atom catalyst (Sn‐NOC) with an asymmetric SnN_3_O_1_ configuration is an efficient and robust CO_2_‐to‐CO conversion catalyst. The Sn‐NOC catalyst shows a maximum CO FE of 94% and a CO partial current density of 13.9 mA cm^−2^ at −0.7 V. Further experimental results and DFT calculations show that the asymmetric atomic interface in SnN_3_O_1_ reduces the energy barrier of rate determining step for CO formation while increasing the energy for *OCO adsorption, the key step for HCOOH production. Therefore, the asymmetric SnN_3_O_1_ structure shows an impressive FE for CO formation and suppresses the formation of HCOOH, the main product for previously reported Sn‐based catalysts. Our work provides an efficient method for controlling the catalytic path and switching the product selectivity of SACs. This approach can be extended to other PCET electrochemical conversions, thus has a wider scope.

## 4 Experimental Section

### Chemicals

Zinc nitrate hexahydrate (Zn(NO_3_)_2_·6H_2_O, AR 99%) and 2‐methyl imidazole (2‐MeIM, AR 98%) were obtained from Aladdin. Stannic oxide (SnO_2_, AR 99.5%) was acquired from RHAWN. Methanol and Ethanol were purchased from Kermel. Nafion solution (5 wt.%) was acquired from Sigma‐Aldrich. Potassium bicarbonate (KHCO_3_) was obtained from Aladdin. All of the chemicals were used without any further purification.

### Synthesis of NC

3.0 g Zn(NO_3_)_2_·6H_2_O and 6.5 g 2‐methyl imidazole were dissolved in 50 and 100 mL methanol, respectively. Both solutions were stirred at 25 °C for 1 h. Then the two solutions were mixed and stirred vigorously for 24 h. During stirring, some precipitates formed and the obtained precipitates were centrifuged and washed three times with methanol, and subsequently vacuum dried at 60 °C overnight, giving ZIF‐8. The as‐prepared ZIF‐8 was heated under Ar at 950 °C for 1 h to obtain NC.

### Synthesis of Sn‐NOC Samples

The SnO_2_ power and the NC were separately placed on the porcelain boat and were heated at 950, 1000, and 1050 °C for 5 h, respectively, under Ar flow.

### Synthesis of SnO_2_‐NC Samples

100 mg SnCl_2_·2H_2_O was dissolved in 20 mL 0.02 M HCl and 40 mg NC was dispersed in 20 mL deionized water, respectively. The solutions were mixed and stirred vigorously for 15 min. The black solid powder was collected and washed with deionized water and dried under vacuum at 80 °C. The powder was then calcined under argon at 300 °C for 2 h to obtain SnO_2_‐NC.

### Characterization

TEM images were carried out on JEOL JEM 2100plus and AC HADDF‐STEM images were gained on JEM‐ARM300F microscope. XRD patterns were collected on the D8 Discover. XPS measurements were performed on ESCALAB 250Xi. HORIBA in Via Reflex was used to perform the Raman measurements with the laser of 532 nm. ICP‐MS was performed on Agilent 5110 to detect mass content of Sn species. AUTO CHEM 2920 was used to obtain TPD patterns.

### Electrochemical Measurements

All electrochemical measurements in this work were performed in a conventional three‐electrode cell using CHI760E electrochemical workstation. 4.0 mg of catalyst was dissolved in mixture solution containing 475 µL of ethanol, 475 µL of ultrapure water, and 50 µL of Nafion solution (5 wt%), and sonicated for 30 min. Then, electrocatalyst ink was dropped on the carbon paper to achieve a loading of 0.6 mg cm^−2^. The compartment of the H‐type electrolytic cell used in the ECRR experiment was separated by an ion exchange membrane, with Ag/AgCl as the reference electrode, platinum wire as the counter electrode, and the 0.1 cm^2^ carbon paper with catalyst as the working electrode. Electrode potentials were converted to the RHE by the followed equation: E (versus RHE) = E (versus Ag/AgCl) + 0.224 V + 0.0596 × pH.

## Conflict of Interest

The authors declare no conflict of interest.

## Supporting information

Supporting Information

## Data Availability

Research data are not shared.

## References

[1] a) Y. Y. Birdja , E. Perez‐Gallent , M. C. Figueiredo , A. J. Gottle , F. Calle‐Vallejo , M. T. M. Koper , Nat. Energy 2019, 4, 732;

[2] a) X. P. Qin , S. Q. Zhu , F. Xiao , L. L. Zhang , M. H. Shao , ACS Energy Lett. 2019, 4, 1778;

[3] a) L.‐H. Zhang , Y. M. Shi , Y. Wang , N. R. Shiju , Adv. Sci. 2020, 7, 1902126; 10.1002/advs.201902126 PMC7055564 32154069

[4] X. L. Zu , X. D. Li , W. Liu , Y. F. Sun , J. Q. Xu , T. Yao , W. S. Yan , S. Gao , C. M. Wang , S. Q. Wei , Y. Xie , Adv. Mater. 2019, 31, 1808135. 10.1002/adma.201808135 30790366

[5] W. F. Xie , H. Li , G. Q. Cui , J. B. Li , Y. K. Song , S. J. Li , X. Zhang , J. Y. Lee , M. F. Shao , M. Wei , Angew. Chem., Int. Ed. 2021, 60, 7382. 10.1002/anie.202014655 33319448

[6] Z. Chen , A. Huang , K. Yu , T. Cui , Z. Zhuang , S. Liu , J. Li , R. Tu , K. Sun , X. Tan , J. Zhang , D. Liu , Y. Zhang , P. Jiang , Y. Pan , C. Chen , Q. Peng , Y. Li , Energy Environ. Sci. 2021, 14, 3430.

[7] H. Shang , X. Zhou , J. Dong , A. Li , X. Zhao , Q. Liu , Y. Lin , J. Pei , Z. Li , Z. Jiang , D. Zhou , L. Zheng , Y. Wang , J. Zhou , Z. Yang , R. Cao , R. Sarangi , T. Sun , X. Yang , X. Zheng , W. Yan , Z. Zhuang , J. Li , W. Chen , D. Wang , J. Zhang , Y. Li , Nat. Commun. 2020, 11, 3049. 32546781 10.1038/s41467-020-16848-8 PMC7297793

[8] B. Zhang , J. Zhang , J. Shi , D. Tan , L. Liu , F. Zhang , C. Lu , Z. Su , X. Tan , X. Cheng , B. Han , L. Zheng , J. Zhang , Nat. Commun. 2019, 10, 2980. 31278257 10.1038/s41467-019-10854-1 PMC6611886

[9] X. Y. Zhao , S. H. Huang , Z. Y. Chen , C. B. Lu , S. Han , C. C. Ke , J. H. Zhu , J. C. Zhang , D. Tranca , X. D. Zhuang , Carbon 2021, 178, 488.

[10] J. Zhang , M. Zhang , Y. Zeng , J. Chen , L. Qiu , H. Zhou , C. Sun , Y. Yu , C. Zhu , Z. Zhu , Small 2019, 15, 1900307. 10.1002/smll.201900307 31058413

[11] Z. K. Yang , B. X. Chen , W. X. Chen , Y. T. Qu , F. Y. Zhou , C. M. Zhao , Q. Xu , Q. H. Zhang , X. Z. Duan , Y. Wu , Nat. Commun. 2019, 10, 3734. 31427572 10.1038/s41467-019-11796-4 PMC6700197

[12] J. P. Wang , G. K. Han , L. G. Wang , L. Du , G. Y. Chen , Y. Z. Gao , Y. L. Ma , C. Y. Du , X. Q. Cheng , P. J. Zuo , G. P. Yin , Small 2018, 14, 1704282. 10.1002/smll.201704282 29504246

[13] W. Ni , Z. Liu , Y. Zhang , C. Ma , H. Deng , S. Zhang , S. Wang , Adv. Mater. 2021, 33, 2003238. 10.1002/adma.202003238 33241569

[14] a) H. Fei , J. Dong , M. J. Arellano‐Jimenez , G. Ye , N. D. Kim , E. L. Samuel , Z. Peng , Z. Zhu , F. Qin , J. Bao , M. J. Yacaman , P. M. Ajayan , D. Chen , J. M. Tour , Nat. Commun. 2015, 6, 8668; 26487368 10.1038/ncomms9668 PMC4639894

[15] H. Fei , J. Dong , C. Wan , Z. Zhao , X. Xu , Z. Lin , Y. Wang , H. Liu , K. Zang , J. Luo , S. Zhao , W. Hu , W. Yan , I. Shakir , Y. Huang , X. Duan , Adv. Mater. 2018, 30, 1802146. 10.1002/adma.201802146 30016001

[16] C. Mattevi , G. Eda , S. Agnoli , S. Miller , K. A. Mkhoyan , O. Celik , D. Mastrogiovanni , G. Granozzi , E. Garfunkel , M. Chhowalla , Adv. Funct. Mater. 2009, 19, 2577.

[17] H. C. Schniepp , J. L. Li , M. J. McAllister , H. Sai , M. Herrera‐Alonso , D. H. Adamson , R. K. Prud'homme , R. Car , D. A. Saville , I. A. Aksay , J. Phys. Chem. B 2006, 110, 8535. 16640401 10.1021/jp060936f

[18] Y. Zhao , J. Liang , C. Wang , J. Ma , G. G. Wallace , Adv. Energy Mater. 2018, 8, 1702524.

[19] P. F. Hou , X. P. Wang , Z. Wang , P. Kang , ACS Appl. Mater. Interfaces 2018, 10, 38024. 30354056 10.1021/acsami.8b11942

[20] L.‐P. Yuan , W.‐J. Jiang , X.‐L. Liu , Y.‐H. He , C. He , T. Tang , J. Zhang , J.‐S. Hu , ACS Catal. 2020, 10, 13227.

[21] W. Ye , Y. Yang , M. Arif , S. Yang , X. Fang , M. A. Mushtaq , X. Chen , D. Yan , ACS Sustainable Chem. Eng. 2020, 8, 15946.

[22] a) D. A. Henckel , M. J. Counihan , H. E. Holmes , X. Chen , U. O. Nwabara , S. Verma , J. Rodríguez‐López , P. J. A. Kenis , A. A. Gewirth , ACS Catal. 2020, 11, 255;

[23] W. Ni , Y. Gao , Y. Lin , C. Ma , X. Guo , S. Wang , S. Zhang , ACS Catal. 2021, 11, 5212.

[24] Y. Lu , H. J. Wang , P. F. Yu , Y. F. Yuan , R. Shahbazian‐Yassar , Y. Sheng , S. Y. Wu , W. G. Tu , G. Y. Liu , M. Kraft , R. Xu , Nano Energy 2020, 77, 105158.

