# Supplementary material for: Control over Electrochemical CO2 Reduction Selectivity by Coordination Engineering of Tin Single‐Atom Catalysts
Source: Adv Sci (Weinh). 2021 Oct 24;8(23):2102884. doi: 10.1002/advs.202102884 (PMC8655193; doi:10.1002/advs.202102884)
Supplement: Supplementary file 1 — Supporting Information [file ADVS-8-2102884-s001.pdf]

© 2021 Wiley-VCH GmbH

ADVANCED SCIENCE
Open Access

Supporting Information

for *Adv. Sci.,* DOI: 10.1002/advs.202102884

Control over electrochemical CO_2 reduction selectivity by coordination engineering of tin single-atom catalysts

Jiangyi Guo, Wenlin Zhang, Lu-Hua Zhang, Datong Chen, Jiayu Zhan, Xueli Wang, N. Raveendran Shiju,* and Fengshou Yu**

Control over electrochemical CO_2 reduction selectivity by coordination engineering of tin single-atom catalysts

Jiangyi Guo, Wenlin Zhang, Lu-Hua Zhang, Datong Chen, Jiayu Zhan, Xueli Wang, N. Raveendran Shiju,* and Fengshou Yu**

J. Guo, Prof. W. Zhang, Dr. L.-H. Zhang, D. Chen, J. Zhan, X. Wang, Dr. F. Yu
Tianjin Key Laboratory of Chemical Process Safety, National-Local Joint Engineering Laboratory for Energy Conservation in Chemical Process Integration and Resources Utilization, School of Chemical Engineering and Technology, Hebei University of Technology, Tianjin 300130, P. R. China.
E-mail: luhuazhang@hebut.edu.cn, yfsh@hebut.edu.cn
Dr. N. R. Shiju
Van 't Hoff Institute for Molecular Sciences
University of Amsterdam
P.O. Box 94157, 1090GD Amsterdam, The Netherlands
E-mail: n.r.shiju@uva.nl

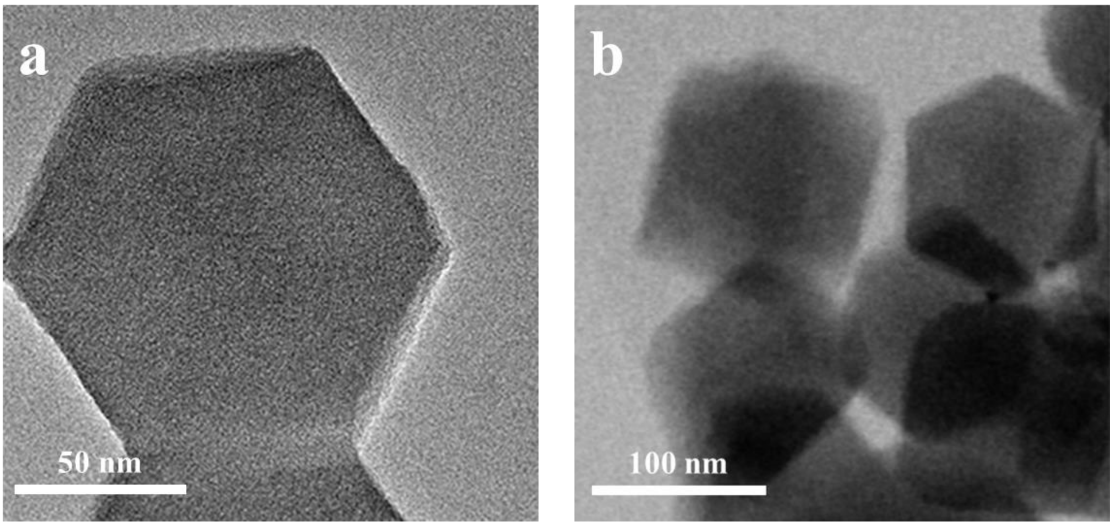

Figure S1. TEM images of (a) NC and (b) Sn-NOC

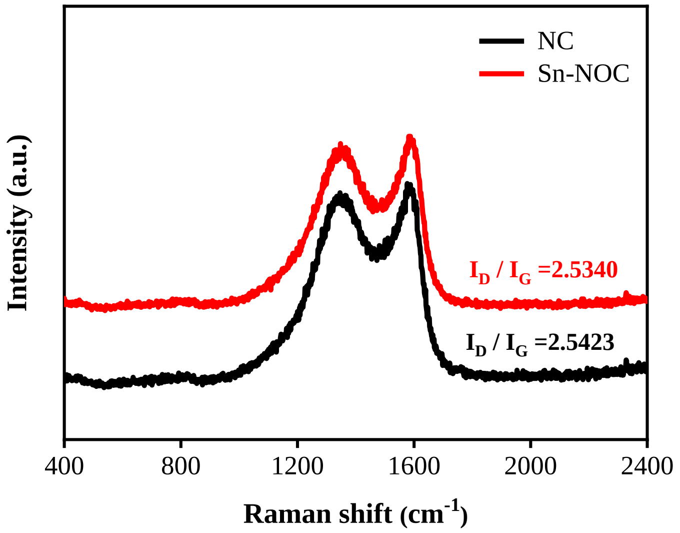

Figure S2. Raman spectra of NC and Sn-NOC

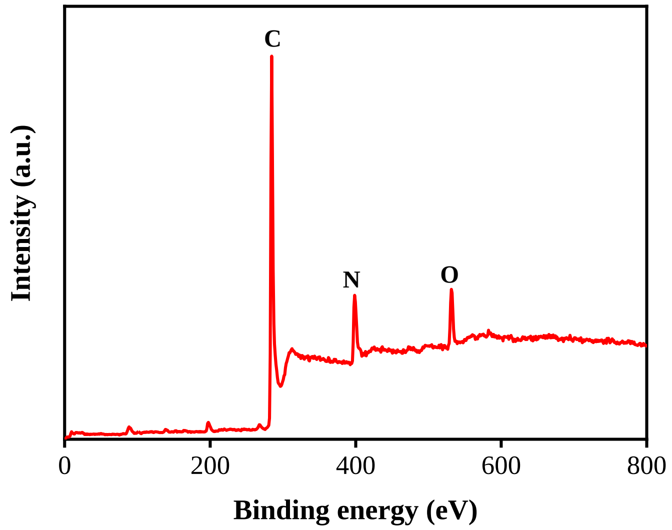

Figure S3. XPS spectrum of NC

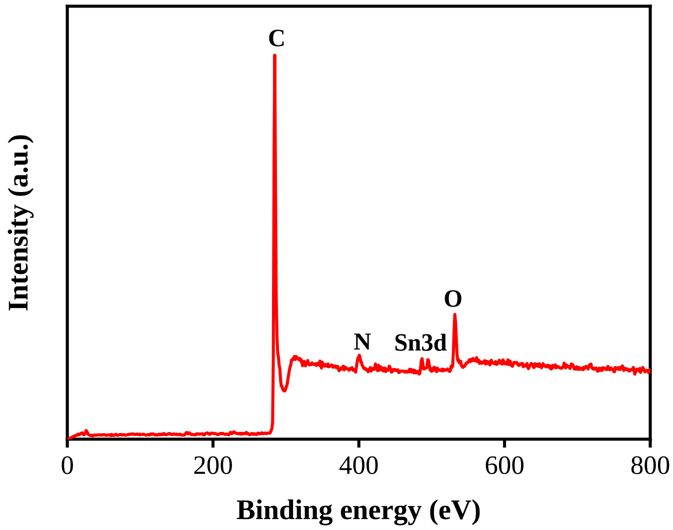

Figure S4. XPS spectrum of Sn-NOC

Table S1. Relative contents of Sn, O, N and C in the NC and Sn-NOC samples estimated from XPS analysis

sample	C (at. %)	N (at. %)	O (at. %)	Sn (at. %)
NC	79.68	14.19	6.13	0
Sn-NOC	84.26	3.88	10.93	0.93

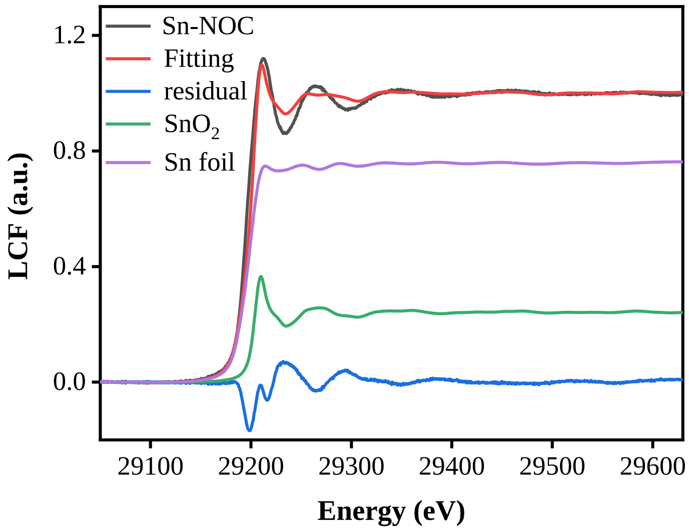

Figure S5. Linear Combination Fitting of XANES data of the Sn-NOC catalyst

Table S2. Linear Combination Fitting of XANES data for Sn-NOC

	Weight of Sn foil	Weight of SnO_2	Averaged Sn valence
Sn-NOC	0.758 (0.039)	0.242 (0.020)	0.968

Table S3. EXAFS data fitting results of the Sn foil, SnO_2, and Sn-NOC

Sample	Shell	CN^a	$R(Å)^b$	$\sigma^2(Å^2)^c$	$\Delta E_0(eV)^d$	R factor
Sn foil	Sn-Sn	4.0*	3.00	0.0049	2.4	0.0056
SnO_2	Sn-O	5.8	2.05	0.0020	2.5	0.0049
SnN_3O_1	Sn-O	1.0*	1.99	0.0002	10.9	0.0096
	Sn-N	3.4	2.08	0.0002		
SnN_2O_2	Sn-O	2.0*	1.99	0.0005	10.6	0.0182
	Sn-N	2.4	2.10	0.0005		

[a]CN, coordination number; [b]R, distance between absorber and backscatter atoms; [c]σ^2, Debye-Waller factor to account for both thermal and structural disorders; [d]ΔE_0, inner potential correction; R factor indicates the goodness of the fit. S_0^2 was fixed to 0.932, according to the experimental EXAFS fit of Sn foil by fixing CN as the known crystallographic value. SnN_3O_1 structure is more reasonable.

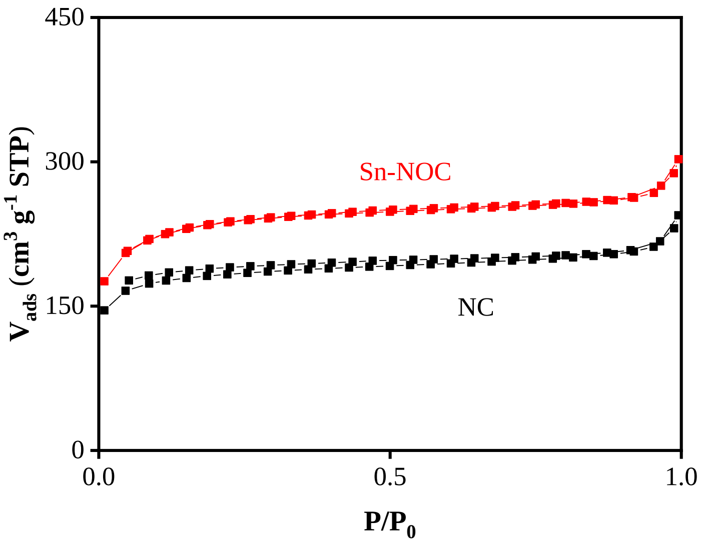

Figure S6. N_2 sorption isotherm curves of the Sn-NOC and NC

Table S4. The structure parameters of NC and Sn-NOC

Sample	S_{BET} (m^2 g^{-1})	S_{mic} (m^2 g^{-1})	V_{total} (cm^3 g^{-1})	V_{mic} (cm^3 g^{-1})
NC	685.6	568.5	0.33	0.28
Sn-NOC	862.1	700.1	0.41	0.31

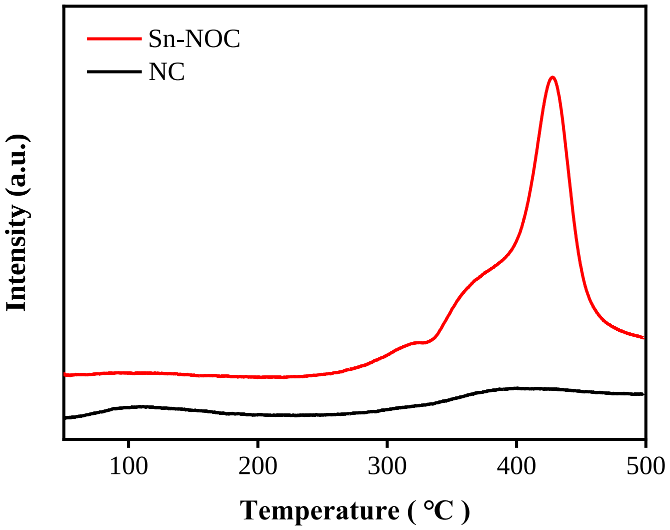

Figure S7. CO_2-TPD of NC and Sn-NOC

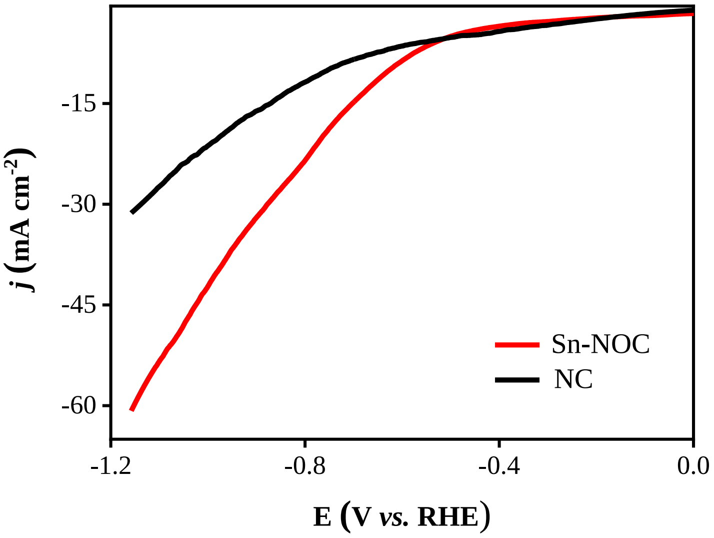

Figure S8. LSV curves of Sn-NOC and NC in CO_2-saturated 0.1 M $KHCO_3$ aqueous solution

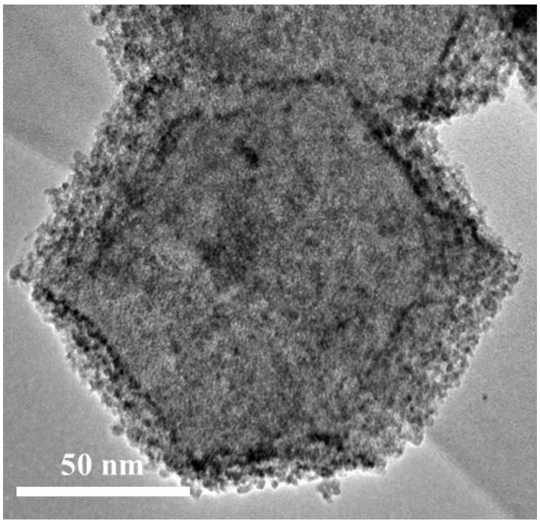

Figure S9. TEM image of SnO_2-NC

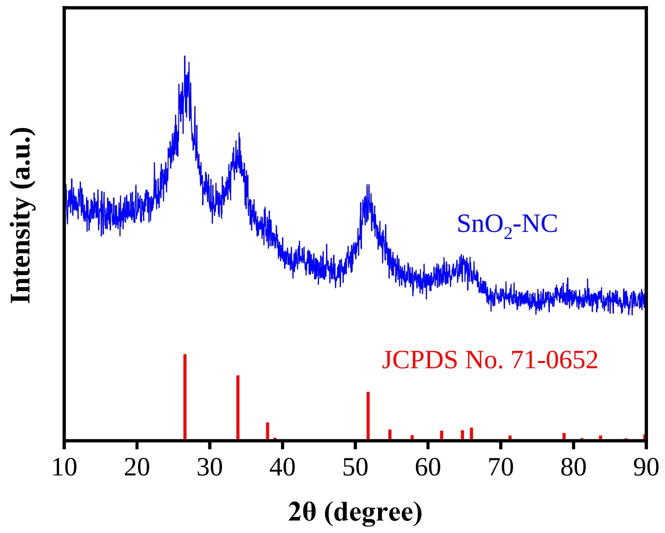

Figure S10. XRD pattern of SnO_2-NC

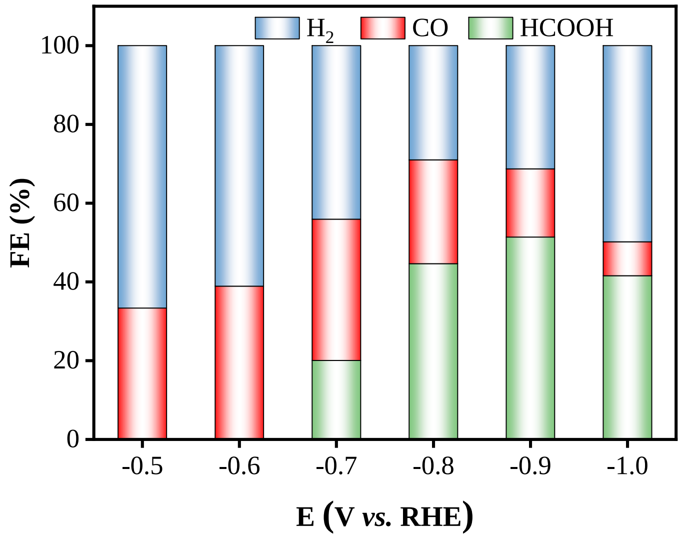

Figure S11. FE of production at various applied potentials of SnO_2-NC

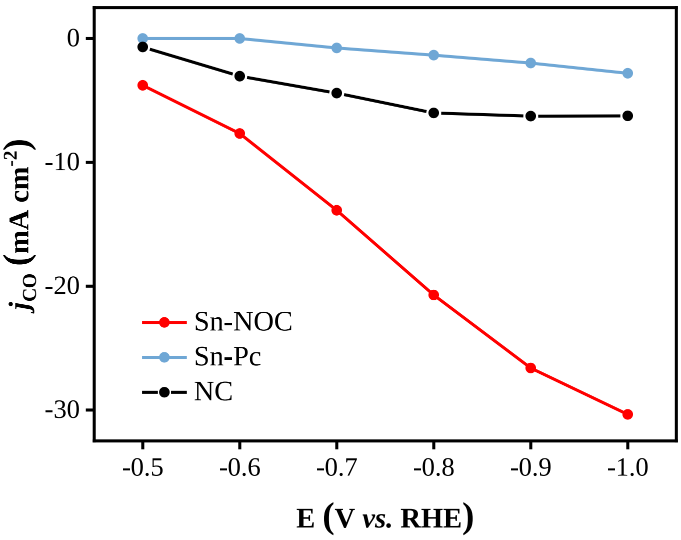

Figure S12. The CO partial current density for Sn-NOC, Sn-Pc and NC

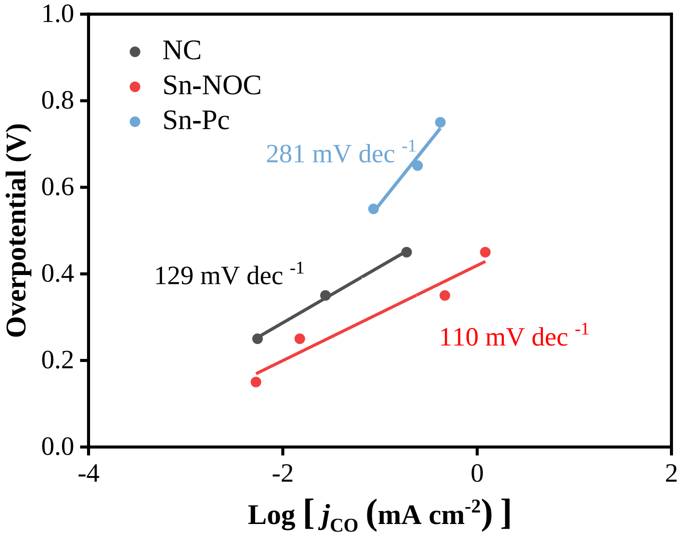

Figure S13. Tafel plots of for Sn-NOC, Sn-Pc and NC

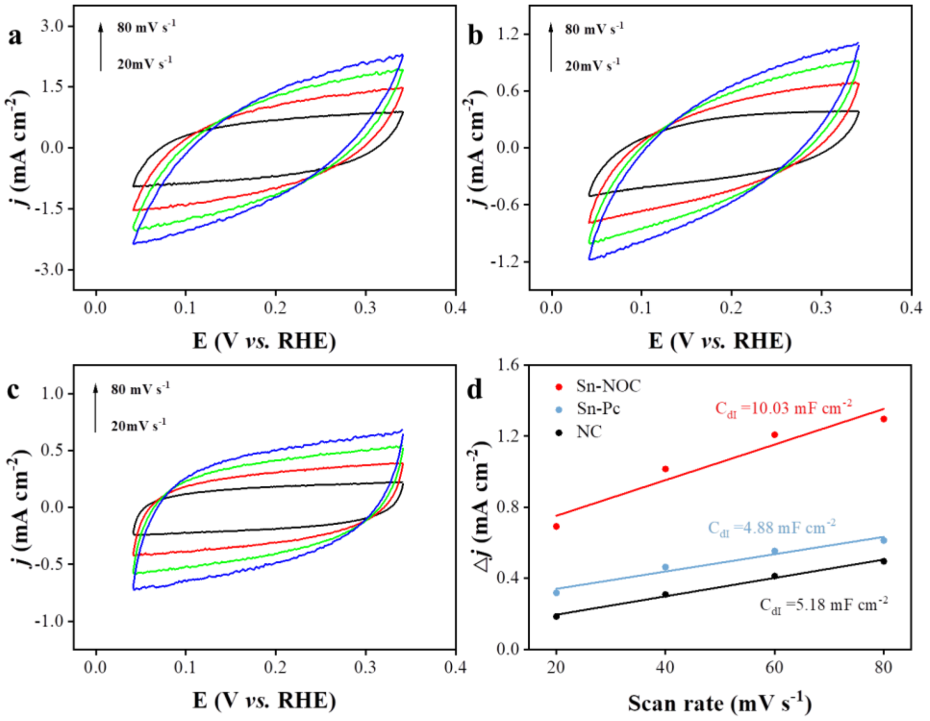

Figure S14. (a-c) CV curves at various scan rates and (d) the corresponding C_{dl} by ECSA of Sn-NOC, Sn-Pc and NC

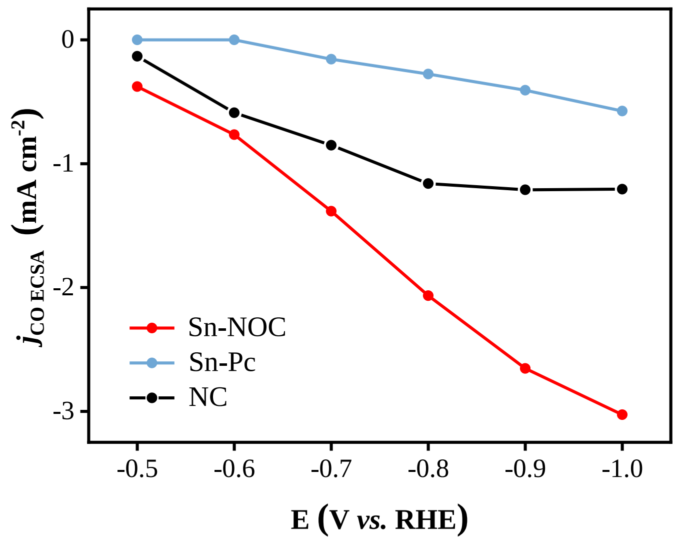

Figure S15. ECSA normalized total current density of Sn-NOC, Sn-Pc and NC

Table S5. TOF (h^{-1}) values of ECRR to CO

Potential (V vs. RHE)	$Sn\text{-}N_3O_1$	$Sn\text{-}N_4$
-0.5 V	2906.5	0
-0.6 V	5899.5	0

-0.7 V	10666.6	15.6
-0.8 V	15929.6	27.6
-0.9 V	20458.5	40.6
-1.0 V	23340.5	57.5

Table S6. Summary of the reported activities of carbon-based SACs for CO_2 electrochemical reduction catalysts

Catalyst	TOF (h^{-1})	Ref.
Sn-NOC	23340.5	This work
NiSn-APC	4752	[1]
NC-CNTs (Ni)	11648	[2]
Ni-N-C	9580.3	[3]
Ni SAs/N-C	5273	[4]
Fe-N/CNF-2	7114	[5]
Co-N_5/HNPCSs	480.2	[6]
Mn-N_4Cl	38347	[7]
Fe_1NC/S_1-1000	2225	[8]

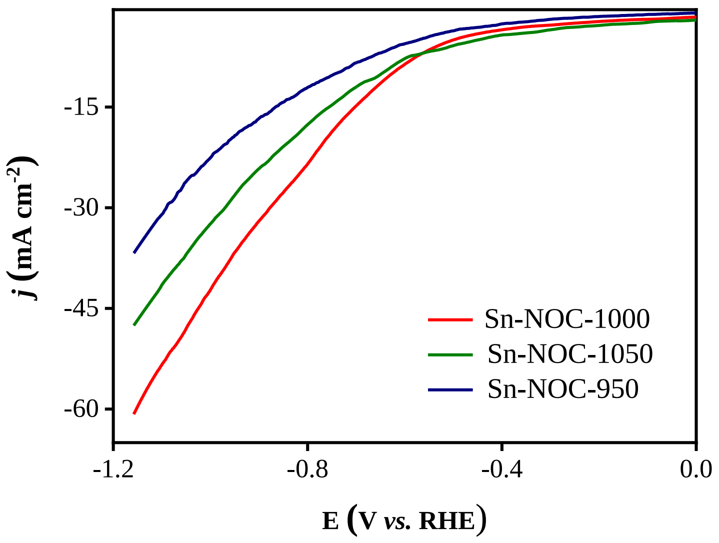

Figure S16. LSV curves of Sn-NOC-x in CO_2-saturated 0.1 M $KHCO_3$ aqueous solution

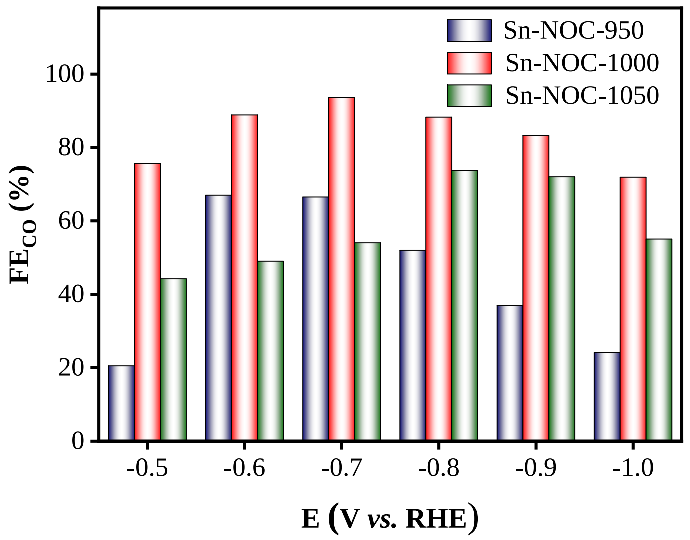

Figure S17. FE of production at various applied potentials of Sn-NOC-x

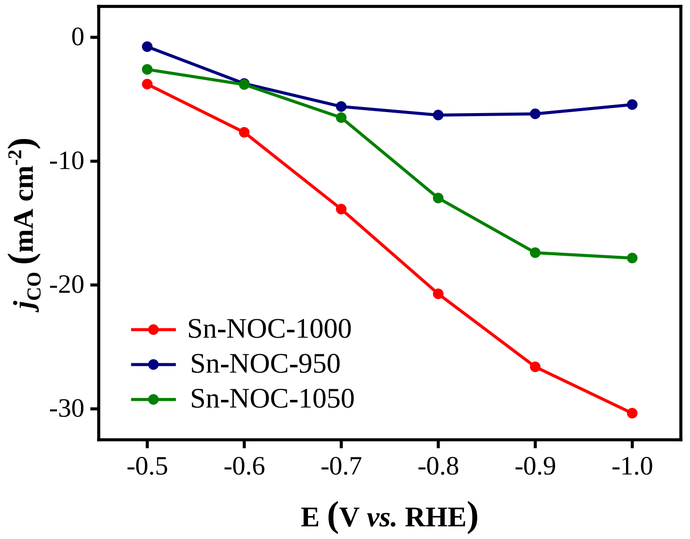

Figure S18. The CO partial current density for Sn-NOC-x

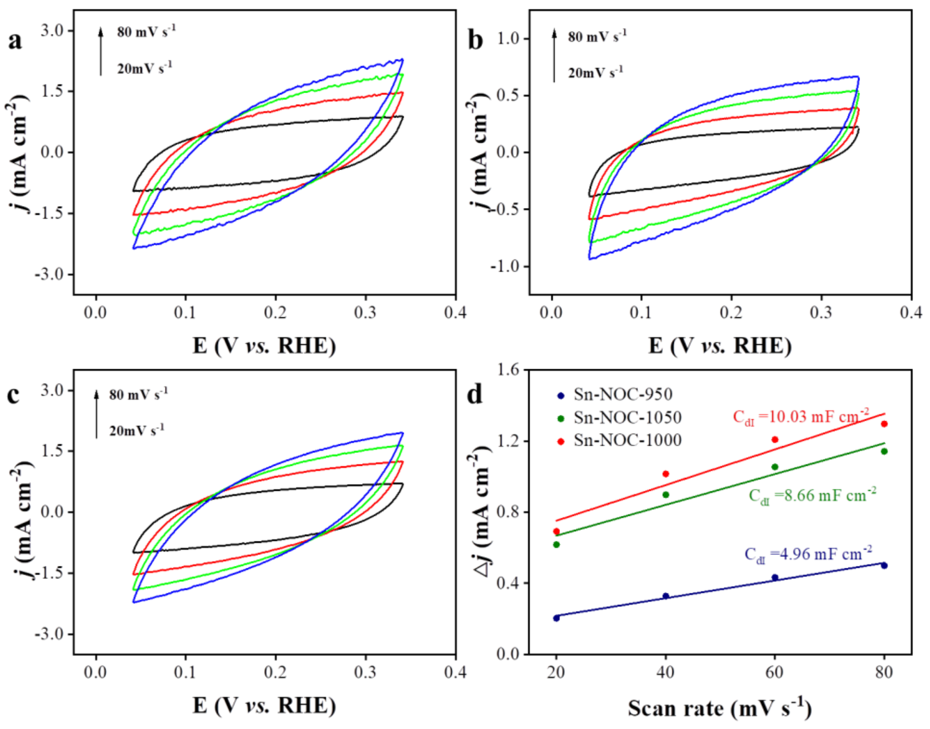

Figure S19. (a-c) CV curves at various scan rates and (d) the corresponding C_{dl} by ECSA of Sn-NOC-x

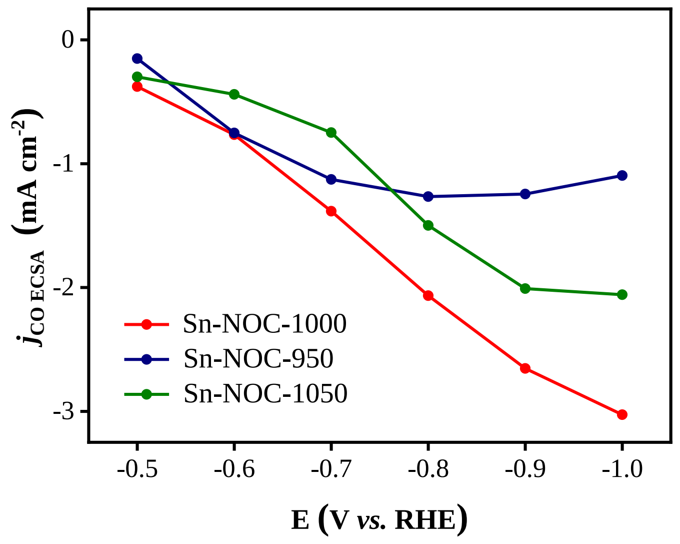

Figure S20. ECSA normalized total current density of Sn-NOC-x

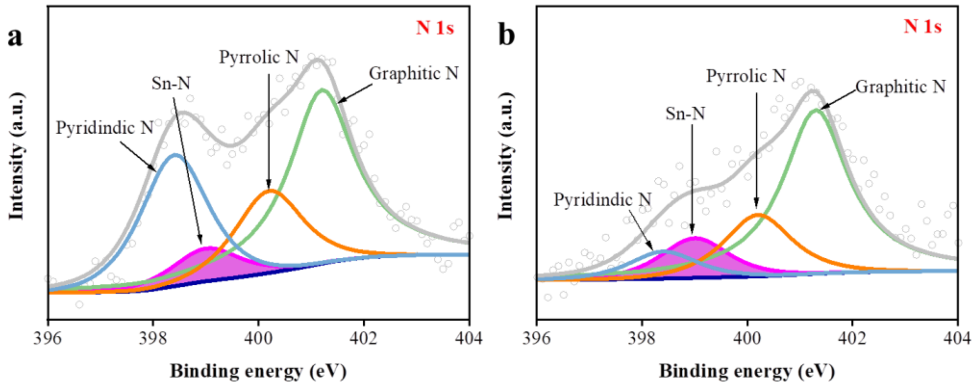

Figure S21. XPS N1s spectra of the (a) Sn-NOC-950 and (b) Sn-NOC-1050 composites

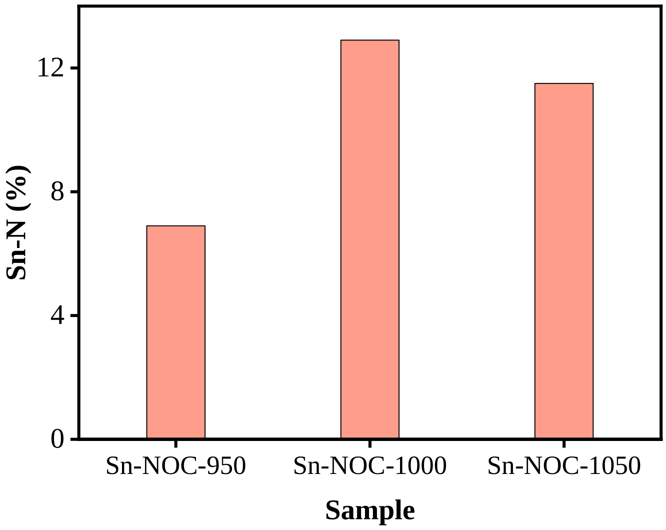

Figure S22. The Sn-N contents of Sn-NOC-x prepared at different pyrolysis temperatures

Table S7. Different N contents from N1s spectra of Sn-NOC-x samples

Sample	Sn-N (at. %)	Pyridinic N (at. %)	Graphitic N (at. %)	Pyrrolic N (at. %)
Sn-NOC-950	6.9	27.6	48.3	17.2
Sn-NOC-1000	12.9	25.8	51.6	9.7
Sn-NOC-1050	11.5	7.8	62.5	18.2

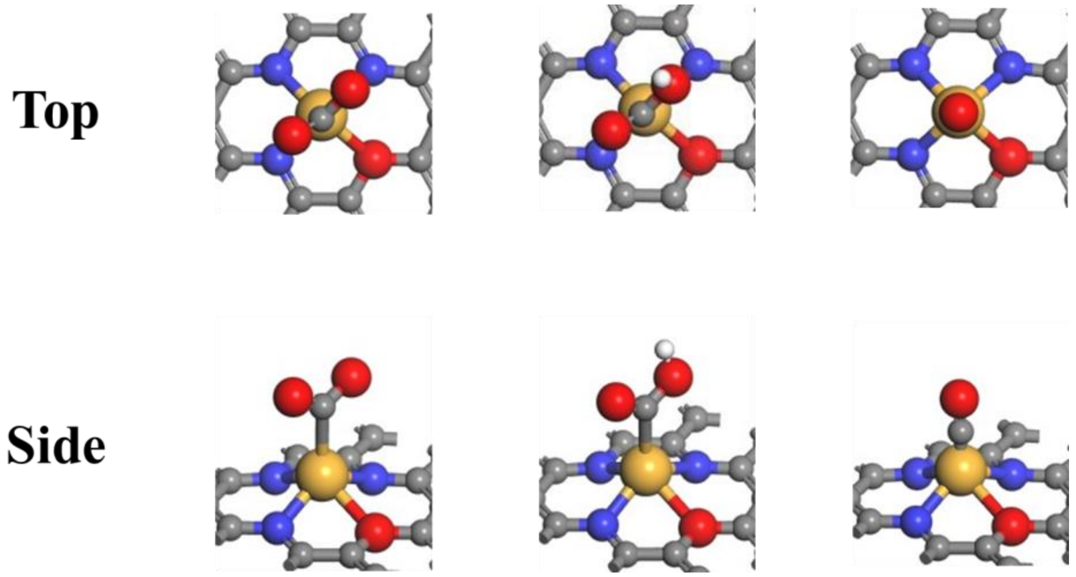

Figure S23. The CO pathway of ECRR on SnN_3O_1

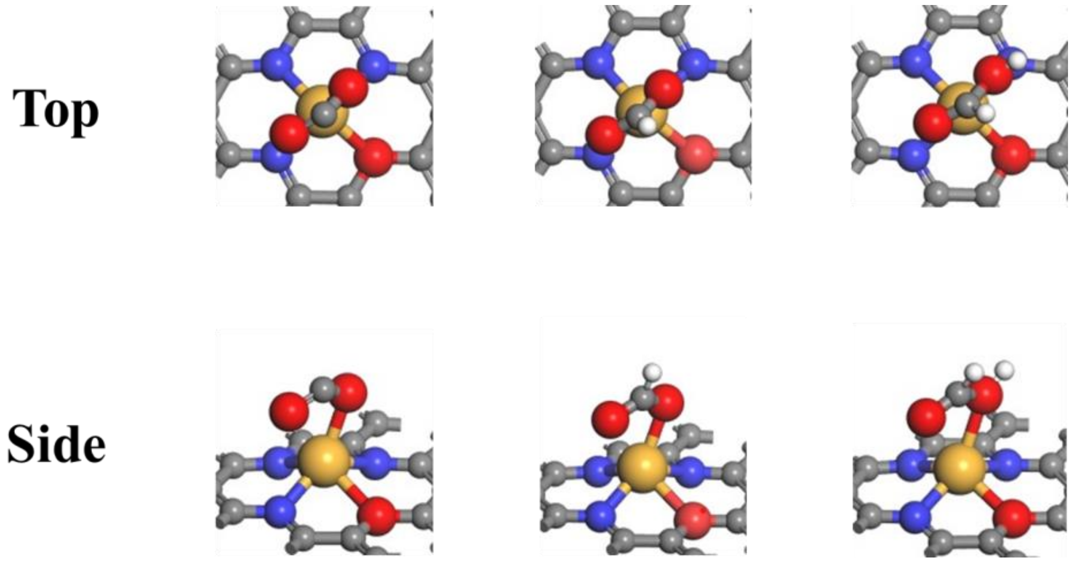

Figure S24. The HCOOH pathway of ECRR on SnN_3O_1

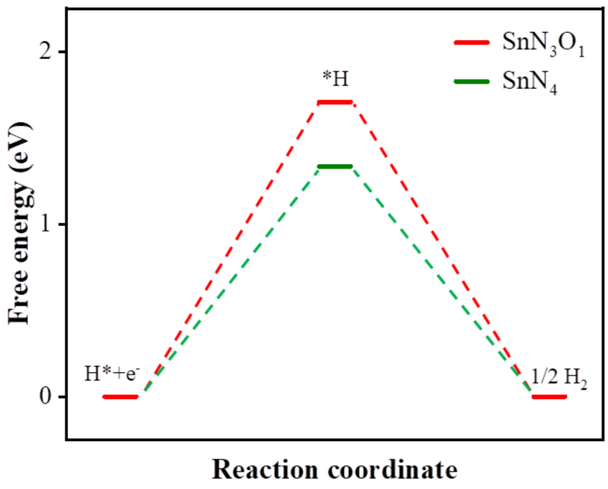

Figure S25. The calculated Gibbs free energy diagrams for the H_2 evolution reaction

References

[1] W. Xie, H. Li, G. Cui, J. Li, Y. Song, S. Li, X. Zhang, J. Y. Lee, M. Shao, M. Wei, *Angew. Chem. Int. Ed.* **2021**, *60*, 7382.

[2] Q. Fan, P. Hou, C. Choi, T. S. Wu, S. Hong, F. Li, Y. L. Soo, P. Kang, Y. Jung, Z. Sun, *Adv. Energy Mater.* **2019**, *10*, 1903068.

[3] X.-M. Hu, H. H. Hval, E. T. Bjerglund, K. J. Dalgaard, M. R. Madsen, M.-M. Pohl, E. Welter, P. Lamagni, K. B. Buhl, M. Bremholm, M. Beller, S. U. Pedersen, T. Skrydstrup, K. Daasbjerg, *ACS Catal.* **2018**, *8*, 6255.

[4] C. M. Zhao, X. Y. Dai, T. Yao, W. X. Chen, X. Q. Wang, J. Wang, J. Yang, S. Q. Wei, Y. Wu, Y. D. Li, *J. Am. Chem. Soc.* **2017**, *139*, 8078.

[5] Q. Cheng, K. Mao, L. Ma, L. Yang, L. Zou, Z. Zou, Z. Hu, H. Yang, *ACS Energy Lett.* **2018**, *3*, 1205.

[6] Y. Pan, R. Lin, Y. Chen, S. Liu, W. Zhu, X. Cao, W. Chen, K. Wu, W. -C. Cheong,

Y. Wang, L. Zheng, J. Luo, Y. Lin, Y. Liu, C. Liu, J. Li, Q. Lu, X. Chen, D. Wang, Q. Peng, C. Chen, Y. Li, *J. Am. Chem. Soc.* **2018**, *140*, 4218.

[7] B. Zhang, J. Zhang, J. Shi, D. Tan, L. Liu, F. Zhang, C. Lu, Z. Su, X. Tan, X. Cheng, B. Han, L. Zheng, J. Zhang, *Nat. Commun.* **2019**, *10*, 2980.

[8] T. Wang, X. Sang, W. Zheng, B. Yang, S. Yao, C. Lei, Z. Li, Q. He, J. Lu, L. Lei, L. Dai, Y. Hou, *Adv. Mater.* **2020**, *32*, 2002430.
